# Researchers' experience with project management in health and medical research: Results from a post-project review

**DOI:** 10.1186/1471-2458-11-424

**Published:** 2011-06-02

**Authors:** Janet M Payne, Kathryn E France, Nadine Henley, Heather A D'Antoine, Anne E Bartu, Elizabeth J Elliott, Carol Bower

**Affiliations:** 1 Population Sciences, Telethon Institute for Child Health Research, Centre for Child Health Research, The University of Western Australia, Roberts Road, Subiaco, 6008, Western Australia, Australia; 2 Centre for Applied Social Marketing Research, Edith Cowan University, Joondalup Drive, Joondalup, 6027, Western Australia, Australia; 3 School of Nursing and Midwifery, Curtin Health Innovation Research Institute, Curtin University of Technology, Kent Street, Bentley, 6102, Western Australia, Australia; 4 Discipline of Paediatrics and Child Health, Sydney Medical School, University of Sydney, University Road, Sydney, 2006, New South Wales, Australia

## Abstract

**Background:**

Project management is widely used to deliver projects on time, within budget and of defined quality. However, there is little published information describing its use in managing health and medical research projects. We used project management in the *Alcohol and Pregnancy Project *(2006-2008) http://www.ichr.uwa.edu.au/alcoholandpregnancy and in this paper report researchers' opinions on project management and whether it made a difference to the project.

**Methods:**

A national interdisciplinary group of 20 researchers, one of whom was the project manager, formed the Steering Committee for the project. We used project management to ensure project outputs and outcomes were achieved and all aspects of the project were planned, implemented, monitored and controlled. Sixteen of the researchers were asked to complete a self administered questionnaire for a post-project review.

**Results:**

The project was delivered according to the project protocol within the allocated budget and time frame. Fifteen researchers (93.8%) completed a questionnaire. They reported that project management increased the effectiveness of the project, communication, teamwork, and application of the interdisciplinary group of researchers' expertise. They would recommend this type of project management for future projects.

**Conclusions:**

Our post-project review showed that researchers comprehensively endorsed project management in the *Alcohol and Pregnancy Project *and agreed that project management had contributed substantially to the research. In future, we will project manage new projects and conduct post-project reviews. The results will be used to encourage continuous learning and continuous improvement of project management, and provide greater transparency and accountability of health and medical research. The use of project management can benefit both management and scientific outcomes of health and medical research projects.

## Background

Project management is described as 'a formalised and structured method for managing change in a rigorous manner' [1]. It is used to 'produce specifically defined deliverables, by a certain time to a defined quality, with a given level of resources, so that planned outcomes and benefits may be achieved' [1]. It is also defined by the Project Management Institute in *A guide to project management body of knowledge: PMBOK guide *[2] (PMBOK) as 'the application of knowledge, skills, tools, techniques to a broad range of activities in order to meet requirements of a particular project' [2]. The PMBOK guide explains project management in terms of five process groups (initiating, planning, executing, controlling and monitoring, and closing), and nine knowledge areas about management (integration, scope, time, cost, quality, resources, communication, risk, and procurement) [2] that are used across the life cycle of a project. It has been suggested that the theory of project management is limited [3,4] and implicit; [5,6] the PMBOK guide provides a rational and analytic approach to project management [7] and is a model that is commonly applied in practice [5].

In general, many projects are not completed on time, within budget, [1,8-10] and to the expected quality [1,11]. However, this may be avoided by the use of project management and an effective project manager who integrates all aspects of the project to ensure its success [1,12]. Project management is used by the military, in engineering, commerce, industry, information systems, financial services, education and training, and health services [13,14]. The Centers for Disease Control and Prevention (CDC) [15] encourage the application of project management in their projects and to rapidly develop solutions in the event of a public health emergency. CDC also provides web-based information and tools to assist project managers and project teams to follow best practices in project management [15].

There are published texts on project management [13] and courses available for researchers [16]. However, not all researchers have received training in project management. Project management could assist in avoiding problems that may arise during research projects such as budget over-runs, missed deadlines and problems with stakeholders [17]. It has been suggested that poor project management is more often responsible for difficulties in health and medical research projects than methodological issues [17].

**Figure 1:**
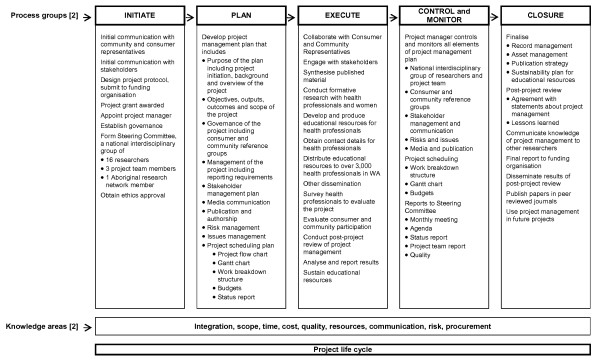
**Project life cycle of the *Alcohol and Pregnancy Project***.

We project managed in the *Alcohol and Pregnancy Project *from 2006-2008. The project, based on our previous research [18] involved providing health professionals (Aboriginal health workers, allied health professionals, community nurses, general practitioners, obstetricians, and paediatricians) in Western Australia (WA) with educational resources to inform them about the prevention of prenatal exposure to alcohol and Fetal Alcohol Spectrum Disorder. We synthesised published material and conducted formative research with health professionals [19] and with Aboriginal and non-Aboriginal women of childbearing age; developed the educational resources and distributed them to over 3,000 health professionals; and surveyed these health professionals to evaluate the project [20]. Figure 1 provides further details of the activities involved in the life cycle of the project.

We used the PMBOK guide [2] project management process groups and knowledge areas across the life cycle of the *Alcohol and Pregnancy Project *(Figure 1) to deliver the project on time, budget and to the required quality. We also used project management to assist our national interdisciplinary group of researchers to use their skills, knowledge and experience to: communicate well; share our understanding of the science, research methods, management of the project and expectations; aid the process of communicating and integrating the project across multiple organisations, professions, consumer and community representatives and stakeholders; and manage the project according to the project protocol and within the allocated budget and three year time frame. The use of project management was a novel approach for researchers involved in this project as none had used it previously.

In this paper we describe project management in the *Alcohol and Pregnancy Project *http://www.ichr.uwa.edu.au/alcoholandpregnancy and report researchers' opinions on the use of project management and whether it made a difference to the project.

## Methods

One researcher (JP), nominated as project manager, attended a one-day intensive workshop on project management fundamentals [21]. The workshop was conducted by the University of Western Australia and was considered appropriate training for employees and researchers who managed large and small projects. The workshop introduced the tools and concepts of project management; the fundamentals and benefits of using the techniques of project management, roles and responsibilities and knowledge of the key competencies and skills of a project manager; and provided an understanding of the impact of project management on project success. The project manager used project management process groups and knowledge areas [2,22] throughout the project to ensure all aspects of the project were planned, implemented, monitored and controlled, and project outputs and outcomes were achieved (Figure 1).

The project was initiated in 2006 when our project grant was awarded. A national interdisciplinary group of 20 researchers-16 investigators, three project team members and a representative of an Aboriginal research network formed the Steering Committee. Most (n = 16) of the researchers were based in WA in the Perth metropolitan area, two were based outside of the metropolitan area, and two were interstate in New South Wales and Queensland (three did not continue to the end of the project).

### Planning the project

The project manager used web-based templates [1] to construct a project management plan which contained nine sections, comprising the following information:

1. The purpose of the plan; details about project initiation, the background and overview of the project.

2. The objectives, outputs and outcomes of the project. The scope of the project was also defined.

3. The management of the project, listed the reporting requirements to the Steering Committee, ethics committees and the funder of the project, and described its governance and specified the roles and name(s) of the:

(a) Project champion (responsible for promoting the benefits of the project to the broader community);

(b) Project leader (assisted with research and project management matters that arose outside of the formal business of the Steering Committee);

(c) Steering Committee (responsible for research and resource decisions essential for the project outputs and outcomes; provided high level advice and expertise to the project team; and ensured appropriate management of the components outlined in the project management plan);

(d) Project team (involved in the practical aspects of the project that were required for the successful delivery of the project outputs and outcomes; it had core members who were joined by other Steering Committee members depending on the stage of the project and the expertise required);

(e) Chairperson (chaired the monthly Steering Committee meetings);

(f) Project manager (responsible for managing the day-to-day aspects of the project, implementing project plans, monitoring progress and budgets through detailed plans and schedules); and

(g) Consumer and community reference groups comprising 13 representatives (provided a forum for participation and consultation and conveyed community perspectives and guidance to enhance the success of the project).

4. The stakeholder management plan and stakeholder groups that employed or set policy directions for health professionals. It also listed groups that provided inputs to the project: those that may contribute to delivery of project outcomes; those that could implement and utilise project outputs and contribute to the achievement of these; those that would be affected by the project outcomes; and those that had potential to positively influence the achievement of project outcomes and support the sustainability of the educational resources. This section also detailed a communication plan for each group of stakeholders incorporating the mode of communication, when this should take place and its content.

5. The media communication and its role in promoting the project as well as educating the community about the adverse effects of alcohol consumption in pregnancy and advocating about issues related to alcohol use during pregnancy.

6. The publication and authorship policy for the project.

7. The risk management plan where the most significant risks to the project were identified, evaluated and prioritised so they could be anticipated, mitigated, and carefully managed to avoid the consequences of project outcomes being delayed or reduced, timeframes extended, costs increased, and the quality of the project diminished. The risks identified were: failure to achieve an outcome, budget over-runs, lack of compliance with Privacy Legislation, and raising anxiety in women of childbearing age about the topic of alcohol use in pregnancy.

8. The issues management plan which committed to monitoring, reviewing and addressing issues or concerns as they arose and reporting these to the Steering Committee.

9. The project scheduling plan comprising a work breakdown structure which detailed all the tasks, the person(s) allocated to the tasks and the commencement and completion times; the project flow chart; and a Gantt chart (condensed from the flow chart) that detailed major tasks against time.

The project management plan was agreed by all members of the Steering Committee at the commencement of the project and was used throughout the project.

### Managing the project

The project manager was responsible for ensuring the management of all the elements of the project management plan. This involved implementing, monitoring and controlling scope creep; collaborating with consumer and community representatives; stakeholder management and communication; risks and issues; media and publication and project scheduling. Meetings were held frequently with the project officer and at least every two weeks with members of the project team.

The Steering Committee supported communication, scientific and quality management aspects of the project. Meetings were held monthly and an agenda and notes from the previous meeting were circulated along with a project status report prepared by the project manager.

The project status report was concise and conveyed essential information about the project. It included an overall summary of the progress of the project; the milestones scheduled for achievement since the previous meeting and performance against these; the milestones scheduled for achievement over the next reporting period; general information; a budget report; a risk management statement specifying any changes to major risks, their likelihood and seriousness and plan for mitigation; an issues report including specific problems and concerns; and recommendations to the Steering Committee.

### Closing the project

The project closure activities included record management, asset management, publication strategy, and a sustainability plan for the educational resources for health professionals. We also conducted a post-project review which is an important part of project management [15] and includes 'lessons learned' [2] from managing the project. The project manager designed a self administered two-page questionnaire comprising 21 questions with both open and closed response options (strongly agree, agree, unsure, disagree, strongly disagree, not applicable). We sought researchers' opinions about project management by incorporating six statements directly from the stated purpose of the project management plan and 15 other questions, 14 of which were adapted from 'lessons learned' [15,23] (Tables 1, 2, 3, 4, 5). In the open ended questions, we asked: what difference the use of project management had made to the project; what worked well; what did not work well; and whether researchers would recommend this type of project management for similar future research projects (Tables 2, 3, 4 and 5).

**Table 1:** Researchers' (n = 15) agreement^a ^with statements about project management

	Strongly agree n (%)	Agree n (%)	Unsure n (%)	Not applicable n (%)
**Statements taken directly from the purpose of the project management plan**				
The use of project management assisted in the delivery of defined project outcomes	7 (46.7)	7 (46.7)	1 (6.7)	0
The use of project management assisted in the process of communicating and integrating project work across multiple organisations and professions	10 (66.7)	5 (33.3)	0	0
The use of project management assisted in clarifying and agreeing goals	10 (66.7)	4 (26.7)	1 (6.7)	0
The use of project management assisted in identifying resources needed for the project	8 (53.3)	4 (26.7)	3 (20.0)	0
The use of project management ensured accountability for results and performance	8 (53.3)	6 (40.0)	1 (6.7)	0
The use of project management fostered a focus on the benefits to be achieved	6 (40.0)	8 (53.3)	1 (6.7)	0
**Statements adapted from 'lessons learned' **[15,23]				
The project manager responded to my questions or comments that were related to the project	12 (80.0)	1 (6.7)	1 (6.7)	1 (6.7)
My expectations were met regarding the frequency and content of information conveyed to me by the project manager	12 (80.0)	3 (20.0)	0	0
The Steering Committee meetings were conducted effectively	8 (53.3)	5 (33.3)	0	2 (13.3)
The format and content of the Project Status Report informed me adequately	8 (53.3)	5 (33.3)	2 (13.3)	0
Project issues were communicated adequately throughout the term of the project	10 (66.7)	3 (20.0)	1 (6.7)	1 (6.7)
Project issues were managed effectively during the project	10 (66.7)	3 (20.0)	2 (13.3)	0
Sufficient time was allocated to review the project outputs (the resources for health professionals)	8 (53.3)	5 (33.3)	2 (13.3)	0
I was satisfied with my involvement in the project	9 (60.0)	6 (40.0)	0	0
The project met my expectations in terms of effort, time and commitment	9 (60.0)	5 (33.3)	1 (6.7)	0

^a ^Possible options: Strongly agree, agree, unsure, disagree, strongly disagree, not applicable

**Table 2:** What difference did the use of project management made to the project?^a^

**Researchers' (n = 15) opinions**
All processes relating to the organisation of the project were streamlined and all involved in the study were kept up to date at all times.
Allowed a good degree of professionalism to develop ensuring that even minor details were accounted for.
Gave everyone the feeling that they were heard and had a valuable contribution.
Kept individuals to task and up to date with each other. Facilitated communication and problem solving and pre-empted and prevented some potential problems occurring.
Allowed efficiency.
I feel that the project management was effective. It enabled the project to be run according to a defined plan and schedule.
I think it made a major contribution towards being a very well organised and professional project.
In this case the project manager has excellent organisational skill so the project management tool in and of itself was really a support document. For less capable and organised project managers this tool would play a more important role.
Organisation of this multifaceted project, specifying and achieving project goals and coordinating staff tasking was facilitated by the explicit use of the project management plan.
The consistent reporting, the clarity of reporting and the attention to all the relevant issues at each step of the project.
I think that it clarified everybody's role in the project. It was an effective way of ensuring the project achieved its research goals, was on time, under budget and produce excellent outputs. It was also important in providing updates of other projects and initiating other research projects.
It enables the process and outputs to be kept up to date. Also kept on track what was at times a complex program of research.
Kept it on track, on budget, effective and efficient.
Supported the establishment and maintenance of teamwork as Steering Committee and project team members were clear on expectations and roles. Established and facilitated effective methods of communication and decision making through regular planned meetings and reporting structures which in turn allowed for expertise within the committee to be utilised and drawn upon easily. Kept the project on track and adhering to the agreed timeframe as milestones were clearly defined.
The project management plan was helpful, probably more so at the beginning of the project when I referred to it often but not as much as the project went on.

^a^Adapted from 'lessons learned' [15,23]

**Table 3:** What was learned about project management that worked well?^a^

**Researchers' (n = 15) opinions**
Clearly defined roles for the key project organisers facilitated all aspects of the study.
Need for pre-arranged and regular meetings and good documentation.
The benefits of inclusion eg. for myself who was not onsite. Despite this I felt there were ample opportunities for me to express any concerns.
Being able to specify who does what and when (timeline) was invaluable to ensuring that tasks were completed in a timely manner.
For me personally it was the accountability of tasks. It did not appear that anything fell off the radar as tasks were allocated and a note kept of the task work and completion.
Objective on-task focus enabled project direction, obstacle avoidance and project completion.
The importance of documentation from start to finish.
Have the project team meet separately and feed into the Steering Committee.
Setting up monthly meetings same time and venue appeared to facilitate attendance by committee members attending from various organisations. Having a very organised project manager was crucial to the success of the project management and this degree of organisation in communication, planning and decision making allowed for committee members to offer the project their expertise despite many other commitments. It was an effective structure to have the project team situated within the Steering Committee. This allowed the project team members to work intensively together on specific tasks and allowed that small group to be equipped with the necessary detailed knowledge of those tasks to make effective decisions on behalf of the Steering Committee. Having a dedicated note-taker was a real asset. This allowed for objective informed detailed and accurate records and fast circulation of notes to committee members.
The constant attention to detail that was documented meticulously and circulated regularly.
The importance of identifying and documenting each step of the project at the beginning and setting timelines and also identifying potential risks.
The project management document provided a sound foundation for managing the project. It highlighted the importance of someone taking on the role of project manager. Spend the time needed to set the project management structure and processes up at the beginning which was done for this project, rather than as you go. Fix dates for meeting-as was done for this project.
Don't know (n = 1).
No response (n = 2).

^a^Adapted from 'lessons learned' [15,23]

**Table 4:** What was learned about project management that did not work well?^a^

**Researchers' (n = 15) opinions**
It is always more difficult being off site than regularly seeing and interacting with the project team but this should not deter inclusion of people off site.
There appeared to be an awful lot of reading for someone who was not directly working on the project.
Dissemination in journal articles has not really happened-perhaps we could have built in more effective plans for that.
Main difficulty for me was the unfamiliarity of this formal project management-will be much better next time it is used but I'm not sure I could use it myself without some formal training.
The committee meeting agendas were very structured. For the most part this facilitated the effectiveness of the meeting in addressing the agenda, however at times may have restricted or prevented the opportunity for open discussion or workshopping of ideas amongst the committee. So while the structured agenda did work well on some aspects I feel that if it was slightly more flexible this would have been valuable also and enhanced input by committee members.
I can't think of anything (n = 3).
Not aware of any (n = 1).
Nothing (n = 1)
Don't know (n = 2).
No response (n = 3).

^a^Adapted from 'lessons learned' [15,23]

**Table 5:** Would you recommend this type of project management for future projects?

**Researchers' (n = 15) opinions**
Yes definitely. It would be difficult to be involved in a study that does not have such an effective project management structure after being part of this project.
Absolutely I am endeavouring to develop the same project management for my own fellowship research project.
Worthwhile model that could be adopted by all projects.
Definitely. Learning about and being involved in this type of project management has been one of the greatest personal outcomes for me in being a part of this project. The lessons learned and experience of this project management will now be a part of my involvement in any future studies.
I think this is a suitable project management approach for projects over a certain funding eg. $150,000. I don't think that I would recommend all of the elements that were used for small projects.
Yes and I have already recommended it!
Yes. I already use it with my research students as a demonstration of how a project should be conducted.
Yes (n = 8).
No response (n = 1).

The questionnaire, a copy of the notes from the Steering Committee meetings and project status reports were sent to researchers remaining on the Steering Committee excluding the project manager (n = 16). The questionnaires were returned to a person who was not a researcher, who de-identified the data before the project manager conducted the analysis. Summary statistics were produced (Table 1) and responses to the open ended questions are reported verbatim (Tables 2, 3, 4, 5).

Approval for the study was obtained from the Women's and Children's Health Services Ethics Committee, the Western Australian Aboriginal Health Information Ethics Committee, and the Edith Cowan University Research Ethics Committee. The ethics committees approved the receipt of a completed questionnaire as evidence of informed consent to participate in the project.

## Results

The project was delivered from 2006-2008 according to the project protocol within the allocated budget and three year time frame. The post-project review questionnaire was returned by 15 of the 16 of the researchers who stayed throughout the term of the project (93.8% response).

### Researchers' agreement with statements about project management

In response to statements taken directly from the purpose of the project management plan and adapted from 'lessons learned', [15,23] the majority of the researchers agreed that the use of project management assisted in the process of communicating and integrating project work across multiple organisations and professions, in clarifying and agreeing goals, in delivering defined project outcomes, and in ensuring accountability for results and performance. The majority also agreed that the format and content of the project status report informed them adequately and that the project met their expectations in terms of effort, time and commitment (Table 1).

### What difference did the use of project management make to the project?

In response to this question, researchers stated that project management had made a difference in terms of communication, teamwork and expertise. They said it 'established and facilitated effective methods of communication and decision making' and 'facilitated communication and problem solving and pre-empted and prevented some problems occurring'. It 'supported the establishment and maintenance of teamwork ... members were clear on expectations and roles'; it ' ... allowed for expertise within the committee to be utilised and drawn upon easily'; and 'allowed for a good degree of professionalism to develop' (Table 2).

Project management was also seen to increase the efficiency and effectiveness of the project. The '... project management was effective. It enabled the project to be run according to a defined plan and schedule' and 'allowed efficiency'. The 'organisation of this multifaceted project, specifying and achieving project goals and coordinating staff tasking was facilitated by the explicit use of the project management plan', '... it clarified everybody's role in the project'. It was 'an effective way of ensuring the project achieved its research goals, was on time, under budget and produce excellent results'. Another difference project management made to the project was '... the consistent reporting, the clarity of the reporting and the attention to all the relevant issues at each step of the project', 'minor details were accounted for' and '...it made a major contribution toward it being a very well organised and professional project' (Table 2).

Project management also made a difference to the project by 'keeping it on track' and keeping members of the Steering Committee 'up to date'. It 'kept on track what was at times a complex program of research'; 'kept it on track, on budget, effective and efficient'; and 'kept the project on track and adhering to agreed timeframe as milestones were clearly defined'. In addition, '...processes relating to the organisation of the project were streamlined and all in the study were kept up to date at all times'; it ' kept individuals to task and up to date with each other'; and it '... enabled process and outputs to be kept up to date' (Table 2).

### What was learned about project management that worked well?

When asked what was learned about project management that worked well, researchers stated 'the project management document provided a sound foundation for managing the project' and commented on the value of the need to 'spend time to set up the project management structure at the beginning which was done for this project, rather than as you go'. Other aspects of project management that worked well included '...the degree of organisation in communication, planning and decision making allowed for committee members to offer the project their expertise'; the '... importance of documentation from start to finish'; and '... constant attention to detail that was documented meticulously'. It also 'highlighted the importance of someone taking on the role of project manager' and provided '... benefits of inclusion' for those who were offsite (Table 3).

Researchers also thought an aspect of project management that worked well was keeping the project 'on task'. An 'objective on-task focus enabled project direction, obstacle avoidance and project completion' and 'accountability of tasks'. Another factor was 'the importance of identifying and documenting each step of the project at the beginning and setting timelines and also identifying potential risks' and 'being able to specify who does what and when (timeline) was invaluable' (Table 3).

Identifying roles of members of the Steering Committee and meeting times was also seen as having worked well. Researchers stated 'setting up monthly meetings same time and venue appeared to facilitate attendance ... it was an effective structure to have the project team situated within the Steering Committee'; ' fix dates for meeting-as was done for this project'; and 'need for pre-arranged and regular meetings and good documentation'. The 'clearly defined roles for the key project organisers facilitated all aspects of the study' (Table 3).

### What was learned about project management that did not work well?

There were very few comments about what did not work well. One researcher stated 'there appeared to be an awful lot of reading', another expressed concern that 'dissemination in journals has not really happened'. Others were not certain about project management '... difficulty for me was unfamiliarity of this formal project management' and '... not sure could do it myself without some formal training'; and the structured nature of the meetings '... the committee meeting agendas were very structured ... more flexible ...would have ... enhanced input by committee members' (Table 4).

### Would you recommend this type of project management for future projects?

Researchers would recommend this type of project management for similar future research projects and thought it was a 'worthwhile model that could be adopted by all projects'. They stated that it would be '... difficult to be involved in a study that does not have such an effective project management structure after being part of this project' and 'learning about and being involved in this type of project management has been one of the greatest personal outcomes for me in being a part of this project'. Others affirmed that 'project management will now be a part of my involvement in any future studies' and that 'I already use it with my research students as a demonstration of how a project should be conducted'. One researcher stated 'I don't think that I would recommend all of the elements that were used for small projects' (Table 5).

## Discussion

The *Alcohol and Pregnancy Project *was completed on time, budget and to the required quality. We found that researchers comprehensively endorsed the use of project management in this research project. Of the 16 researchers remaining in the project, 15 (93.8%) responded to the questionnaire. All agreed that the project management plan had achieved its purpose and would recommend this type of project management for future projects, with some already starting to use it. The majority agreed that the project met their expectations in terms of effort, time and commitment. They found that the use of project management assisted in the process of communicating and integrating project work across multiple organisations and professions, in clarifying and agreeing goals, in assisting in the delivery of defined project outcomes, and in ensuring accountability for results and performance. Project management worked well because it 'provided a sound foundation for managing the project', increased the 'efficiency and effectiveness of the project' and made a difference to communication, teamwork and application of the interdisciplinary group of researchers' expertise.

There are some limitations to these results. They are based on a small number (n = 15) of responses to our post-project review. Bias may have been introduced if researchers were reluctant to criticise project management. Disapproval of project management may have been seen as risking collegial relationships and future projects, [24] and researchers may have been hesitant to acknowledge any failure of project management [24] as they were responsible for ensuring appropriate management of the project. This potential bias is partly overcome by the anonymity of the questionnaire and its transcription by a person who was not one of the researchers. However, the project manager designed the questionnaire and analysed the responses so it is possible that bias may have been introduced. Although selecting a person with no affiliation with the project [25,26] would have been a strength, this does not always happen in practice [24].

Von Zedtwitz (2002) [24] reported from a study of 63 directors and managers of different organisations who had on average been involved in 33 projects, that only 9.5% of post-project-reviews were conducted by external facilitators and only 19% of projects were followed up with a post-project review. Other authors agree that post-project reviews of project management are often not conducted in practice [27-30]. There is a lack of standardised methodology, [29] they are poorly documented, [28] the results are poorly disseminated [27-29] and knowledge gained is not passed on to others for the benefit of future projects [9,26] or to encourage continuous learning [31] and continuous improvement of project management [30].

In the context of this project, it was not possible to conduct a randomised controlled trial to provide a high level of evidence, nor did we have a comparison group. We do not know how the project would have progressed without the use of project management and whether some of the perceived benefits may also have been achieved without its use. A researcher who responded to the questionnaire commented on the excellent organisational skill of the project manager and it is possible that the competency of the project manager may have influenced the results. Project managers' competencies have been considered [1,7,12,32,33] as influencing the success of projects and that project success may depend on the 'right combination of skills and the will of the people involved' [34]. Unfortunately, we did not ask researchers whether factors other than project management had influenced the outcome of the project.

The national interdisciplinary group of researchers who formed the Steering Committee included those with experience in social marketing and behavioural research; public health and health promotion research and epidemiology; health promotion practice, training and education; alcohol and drug research policy and planning; child and Aboriginal health policy and planning; population health policy and planning; paediatric surveillance, dysmorphology and diagnosis; health services delivery and management and expertise in consumer and community participation. It is reported that when collaborators combine their knowledge, experience and skills they have potential to create a whole that may be greater than the sum of the parts [35]. Interdisciplinary research may also assist knowledge generation and transfer [36-38] and aid sustainability, [39] although it may require more time, effort and commitment, [40] resources, [35] and increased communication processes [41]. Our post-project review showed that the use of project management supported effective collaboration of our national interdisciplinary group of researchers. The collaboration was based on equity, [39] with all members able to contribute to discussions, knowledge could be gained from the interdisciplinary group, capacity could be built within the group, and communication and consensus decision making achieved to support the science and management of the project. Researchers reported valuing the contribution that project management made to the efficiency, effectiveness and organisation of the project by allowing the project 'to be run according to a defined plan and schedule'; 'keeping it on track'; 'streamlined' and 'ensuring the project reached its research goals, was on time, under budget and produce excellent outputs'. They reported that project management 'facilitated effective methods of communication and decision making' and that everyone was 'heard and had a valuable contribution'. None of the researchers had previously been involved in project management but all agreed they would recommend project management for future projects. Some appeared to gain insight from being involved in this project and have since project managed other health and medical research projects.

A robust indicator of project management success is the delivery of a project on time, budget and to the required quality. Similarly to other health and medical research projects, the timeframe and budget of this project were limited and proficient project management was required during the life cycle of the project (Figure 1). Given the amount of activities involved in the project, a possible outcome may have been cost and budget over-runs [8-11] and poor quality outputs. The results or our post-project review show that researchers acknowledged the contribution of project management in achieving the high quality project outputs that were on time and budget. This is important because failure to deliver projects on time, budget, and quality represents a poor return on funds (often, as in this case, from the public purse) that are invested in health and medical research. Researchers have a responsibility to avoid waste from poorly produced and disseminated research [42]. Chalmers and Glasziou [42] have estimated that approximately 85% of research funds may be wasted. Although they did not state specifically that project management could reduce this waste, we suggest that a proportion may be reduced if project management was used more frequently in health and medical research projects.

Based on our experience with project management in this project, we will use project management in future research projects and use similar methods to those described in this paper to conduct a post-project review. We now have baseline data for comparison with new projects. The results will be used to encourage continuous learning [31] and continuous improvement of project management, [30] and provide greater transparency and accountability of health and medical research.

## Conclusions

The use of project management in the *Alcohol and Pregnancy Project *http://www.ichr.uwa.edu.au/alcoholandpregnancy facilitated our successful interdisciplinary research activity and delivery of the project according to the project protocol within the allocated budget and three year time frame. Researchers comprehensively endorsed project management and agreed that it had contributed substantially to the research. The use of project management can benefit both management and scientific outcomes of health and medical research projects. We recommend project management to other researchers involved in health and medical research.

### Ethics approval

Approval for the study was obtained from the Women's and Children's Health Services Ethics Committee, the Western Australian Aboriginal Health Information Ethics Committee, and the Edith Cowan University Research Ethics Committee. The ethics committees approved the receipt of a completed questionnaire as evidence of informed consent to participate in the project.

## Declaration of competing interests

The authors declare that they have no competing interests.

## Authors' contributions

All authors have read and approved the final manuscript. JM Payne, N Henley, HA D'Antoine, AE Bartu, E Elliott and C Bower originated the study. All authors contributed to conceptualising ideas, interpreting findings, and reviewing drafts of the article. JM Payne supervised all aspects of its implementation, completed the quantitative analysis, led the writing and completed the first draft. KE France was project officer and C Bower supervised the quantitative analysis.

## Pre-publication history

The pre-publication history for this paper can be accessed here:

http://www.biomedcentral.com/1471-2458/11/424/prepub
